# The karyotypes and evolution of ZZ/ZW sex chromosomes in the genus *Characidium* (Characiformes, Crenuchidae)

**DOI:** 10.3897/CompCytogen.v12i3.28736

**Published:** 2018-10-02

**Authors:** Marcela Baer Pucci, Viviane Nogaroto, Luiz Antonio Carlos Bertollo, Orlando Moreira-Filho, Marcelo Ricardo Vicari

**Affiliations:** 1 Departamento de Genética e Evolução, Universidade Federal de São Carlos, Rodovia Washington Luís, Km 235, 13565-905, São Carlos, São Paulo State, Brazil Universidade Federal de São Carlos São Carlos Brazil; 2 Departamanento de Biologia Estrutural, Molecular e Genética, Universidade Estadual de Ponta Grossa, Av. Carlos Cavalcanti, 4748, 84030-900, Ponta Grossa, Paraná State, Brazil Universidade Estadual de Ponta Grossa Ponta Grossa Brazil

**Keywords:** Chromosomal differentiation, Cryptic species, Repetitive DNA, Speciation genes

## Abstract

Available data on cytotaxonomy of the genus *Characidium* Reinhardt, 1867, which contains the greatest number of species in the Characidiinae (Crenuchidae), with 64 species widely distributed throughout the Neotropical region, were summarized and reviewed. Most *Characidium* species have uniform diploid chromosome number (2n) = 50 and karyotype with 32 metacentric (m) and 18 submetacentric (sm) chromosomes. The maintenance of the 2n and karyotypic formula in *Characidium* implies that their genomes did not experience large chromosomal rearrangements during species diversification. In contrast, the internal chromosomal organization shows a dynamic differentiation among their genomes. Available data indicated the role of repeated DNA sequences in the chromosomal constitution of the *Characidium* species, particularly, in sex chromosome differentiation. Karyotypes of the most *Characidium* species exhibit a heteromorphic ZZ/ZW sex chromosome system. The W chromosome is characterized by high rates of repetitive DNA accumulation, including satellite, microsatellite, and transposable elements (TEs), with a varied degree of diversification among species. In the current review, the main *Characidium* cytogenetic data are presented, highlighting the major features of its karyotype and sex chromosome evolution. Despite the conserved karyotypic macrostructure with prevalent 2n = 50 chromosomes in *Characidium*, herein we grouped the main cytogenetic information which led to chromosomal diversification in this Neotropical fish group.

## Introduction

Crenuchidae (Teleostei: Characiformes) include 18 genera and 95 species (Eschmeyer et al. 2018), grouped in Crenuchinae and Characidiinae (Buckup 1999). *Characidium* Reinhardt, 1867 is the most species-rich genus of Characidiinae, containing 64 valid species, which are morphologically very similar (Buckup 1993), and broadly distributed across the Neotropical region (Eschmeyer et al. 2018). These fishes are small-sized, reaching 15 cm of length at adulthood (Buckup 1999), and some are commercially used in aquarium hobbies. They usually live in streams and can be found in both lentic and lotic habitats (Buckup 1999). Their elongated body shape and ventrally extended pectoral and pelvic fins enable them to attach tightly to the substrate, allowing them to resist to the water flow and capture food (Aranha et al. 2000). *Characidium* can be classified as autochthonous and insectivorous (Aranha et al. 2000, Bastos et al. 2013, Fernandes et al. 2017) and usually do not exhibit morphological sexual dimorphism (Buckup 1999). *Characidiumsatoi* Melo & Oyakawa, 2015 is an exception, where males develop a seasonal darker and uniform pigmentation of the body and head vs. the vertical bars exhibited in females (Melo and Oyakawa 2015).

Phylogenetic analysis removed these fishes from the Characidae along with the Crenuchinae, and this group was organized in a new monophyletic family, the Crenuchidae (Buckup 1998). Phylogenetic relationships are available for most taxa in this family (Buckup 1993). According to available molecular and morphological data, *Characidium* is a monophyletic group, and its most recent common ancestor (Crenuchidae) likely originated during the Eocene, approximately 50.2 Mya. The geological events during this period boosted South American ichthyofauna diversity (Poveda-Martínez et al. 2016).

Based on morphological data, *Characidiumzebra* Eigenmann, 1909 is the most ancestral species of the genus as well as also of Characidiinae (Buckup 1993). An integrative study using cytogenetic data combined to partial *Cytochrome oxidase C subunit 1* (*COI*) and *Cytochrome B* sequences (*Cyt B*) for molecular phylogenetic analyses was applied in some *Characidium* species (Pansonato-Alves et al. 2014). This analysis proposed *Characidium* into two main groups of species: *i)* those which do not exhibit sex chromosomes heteromorphism; and *ii)* those with a ZZ/ZW sex chromosome heteromorphism with a partial or total heterochromatinization of the W chromosome (Pansonato-Alves et al. 2014). In addition, these data suggested: *i)* that the origin of sex chromosomes in analyzed *Characidium* species was unique and considered an apomorphic state and; *ii)* that B chromosomes present in some *Characidium* species presumably showed independent origins (Pansonato-Alves et al. 2014).

Another common characteristic in cytogenetic data of *Characidium* is the occurrence of cryptic species (Vicari et al. 2008, Machado et al. 2011, Pucci et al. 2014). This is suggested to be due to some populations of the same nominal taxa carrying the Z and W chromosomes at different stages of differentiation and apparent flow gene isolation (Vicari et al. 2008). Hence, new *Characidium* species are frequently described in the scientific literature (Melo and Oyakawa 2015, Zanata and Camelier 2015, Zanata and Ohara 2015) and, the genus needs a critical revision.

## General chromosomal characteristics in Characidium

**Figure 1.**
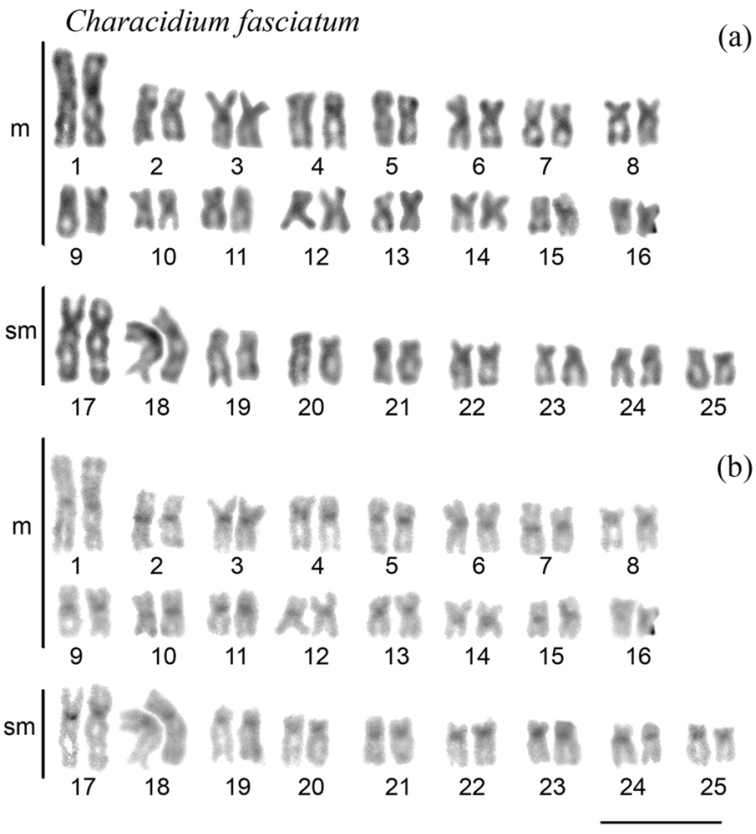
Representative karyotype of *Characidiumfasciatum* with 2n = 50 chromosomes. Cytogenetic data revealed 32 m + 18 sm, without heteromorphic sex chromosomes: **a** conventionally Giemsa-stained **b** sequentially C-banded chromosomes. Scale bar: 5 µm.

Table 1 summarizes the recognized *Characidium* individuals/populations with cytogenetic data. The first cytogenetic investigation of this genus was performed by Miyazawa and Galetti (1994), who analyzed four species and some populations of C.cf.zebra, *Characidium* sp., Characidiumcf.lagosantensis Travassos, 1947 and *Characidiumpterostictum* Gomes, 1947, all of which had 2n = 50 chromosomes (Table 1). In fact, phylogenetically basal *C.zebra*, already possesses such chromosomal plesiomorphic features in the genus (2n = 50; 32m + 18sm), including the absence of heteromorphic sex chromosomes (Vicari et al. 2008, Machado et al. 2011, Pazian et al. 2013). This karyotype pattern occurs in most *Characidium* species (Table 1, Fig. 1), although rare spontaneous triploids have been detected among specimens of *Characidiumgomesi* Travassos, 1956 (Centofante et al. 2001) and C.cf.zebra (Pansonato-Alves et al. 2011a). The evolutionary history of this genus revealed no large chromosomal rearrangements (Machado et al. 2011, Pucci et al. 2014, Scacchetti et al. 2015a, 2015b). However, occasional changes in the karyotypic formula can be found due to differences in the autosome morphology (Table 1).

Interstitial telomeric sites (ITS), which are usually correlated with chromosomal fusions, were identified in the karyotypes of *Characidiumschubarti* Travassos, 1955, *Characidiumlanei* Travassos, 1967, *Characidiumlauroi* Travassos, 1949, *Characidiumtimbuiense* Travassos, 1946, *Characidiumserrano* Buckup & Reis, 1997, and two populations of *C.pterostictum* (Scacchetti et al. 2015c). The varied locations of ITS regions in the karyotypes were ascribed to their probable association with satellite DNA through transposition events and ectopic recombinations (Scacchetti et al. 2015c).

Generally, the constitutive heterochromatin has a preferential distribution in the pericentromeric regions in the most *Characidium* chromosomes, but some large interstitial and terminal blocks were also observed. Chromosomal mapping of 18S and 5S rDNAs showed varied autosomal positions among *Characidium* genomes, ranging from single to multiple sites (Table 1). Nucleolar organizing regions (NORs) were probably related to the origin of the ZZ/ZW sex chromosome system that characterizes many *Characidium* species (Table 1), as commented below.

### Distribution of repetitive DNAs in the Characidium genome

In fishes, tandem or dispersed repetitive DNA sequences are relevant markers for clarifying karyotype evolution and sex chromosome differentiation (Schemberger et al. 2011, Barbosa et al. 2017, do Nascimento et al. 2018, Glugoski et al. 2018). Their accumulation is a key factor for the morphogenesis and the differentiation process of sex chromosomes, and the induction of gene erosion (Matsunaga 2009, Schemberger et al. 2014, Ziemniczak et al. 2014).

**Figure 2.**
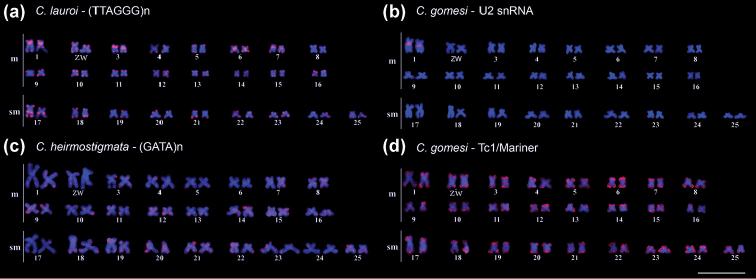
Fluorescence *in situ* hybridization using distinct classes of repeated DNA sequences as probes: In **a** karyotype of *C.lauroi* submitted to (TTAGGG)*_n_* probing (red) **b** karyotype of *C.gomesi* evidencing U2 snRNA sites (red) **c** Karytype of *C.heirmostigmata* submitted to (GATA)*_n_* probing (red) and **d** karyotype of *C.gomesi* evidencing Tc1/Mariner mapping (red). Scale bar: 10 µm.

Despite the highly conserved karyotype structure, the genomes of *Characidium* species display a dynamic pattern of their internal chromosomal composition (Table 1, Fig. 2). Phylogenetics studies using mitochondrial DNA in *Characidium* were used to anchor a comparative cytogenetic analysis using telomeric DNA probe. This data indicated that the ITS signals found in genomes of some *Characidium* species (Fig. 2a) do not have relation with chromosome fusions but, on contrary, are associated with repetitive DNAs dispersion (Scacchetti et al. 2015c). Probably the ITS have origin in the evolutionary lineage of the genus in related hydrographic drainages (Scacchetti et al. 2015c), although some relationship species, such as *C.zebra* and *C.gomesi*, do not harbor such sequences. U2 small nuclear RNA (*snRNA U2*) had a highly conserved distribution in the first m pair in the most species (Fig. 2b), except for *Characidium* sp. aff. *Characidiumvidali* Travassos, 1967, *Characidium* sp. 1 and *Characidiumalipioi* Travassos, 1955, in which *snRNA* U2 site was located in the first submetacentric (sm) pair (Scacchetti et al. 2015b, Serrano et al. 2017).

Distinct microsatellites also had a wide distribution in autosomal pairs (Fig. 2c), probably due to their association with TEs (Scacchetti et al. 2015b, Pucci et al. 2016), such as Tc1-Mariner (Fig. 2d). This pattern was also corroborated by Serrano et al. (2017), evidencing (CA)_15_ and (GA)_15_ autosomal accumulation in the *C.alipioi* genome, as well as of several other microsatellites in *C.zebra* and *C.gomesi*. The molecular characterization and chromosome mapping of the histone genes H1, H3 and H4 were described for *C.zebra* and *C.gomesi* (Pucci et al. 2018). These three histone sequences appear to be associated with TEs and, *in situ* localization, revealed that they are dispersed throughout the autosomes, but they are not involved in the differentiation of the specific region of the W sex chromosome in *C.gomesi* (Pucci et al. 2018).

The available data point to the substantial role of repeated DNA sequences in the chromosomal constitution of *Characidium* species. However, due to the extension of the existing repetitive elements, additional investigations must address their significance in the evolutionary history of *Characidium* and, particularly, in sex chromosome differentiation.

## Supernumerary and sex chromosomes in Characidium

Several Neotropical fish species are carriers of supernumerary or B chromosomes (Carvalho et al. 2008). Additionally, due to the variety of simple or multiple sex chromosome systems in these fishes, differentiated karyotypes exist between sexes (Moreira-Filho et al. 1993, Almeida-Toledo et al. 2001).

B chromosomes, ranging from one to four chromosomes, were described in several *Characidium* species (Table 1). They are hypothesized to have different and independent origins in evolutionary history of the species. To explain the origin, frequency and evolution of B chromosomes it was hypothesized that these elements are derivate from autosomes followed by gene silencing, heterochromatinization, and accumulation of repetitive DNA and transposons (Camacho et al. 2000, Vicari et al. 2011). In some species, B chromosomes are related to sex chromosomes due to share the same repetitive elements (Scacchetti et al. 2015a). In fact, genomes of *C.gomesi*, *C.pterostictum* and *Characidium* sp. aff. *C.vidali* displayed similar repetitive DNA sequences among B and sex chromosomes (Pansonato-Alves et al. 2014, Pazian et al. 2014, Scacchetti et al. 2015a, Serrano et al. 2016), while *Characidiumoiticicai* Travassos, 1967 and *C.alipioi* did not show such shared sequences (Pansonato-Alves et al. 2014, Serrano et al. 2017, respectively). Despite their molecular homology, it was demonstrated that B and W chromosomes do not form multivalent pairings during meiosis in male and female *C.gomesi* individuals.

**Figure 3.**
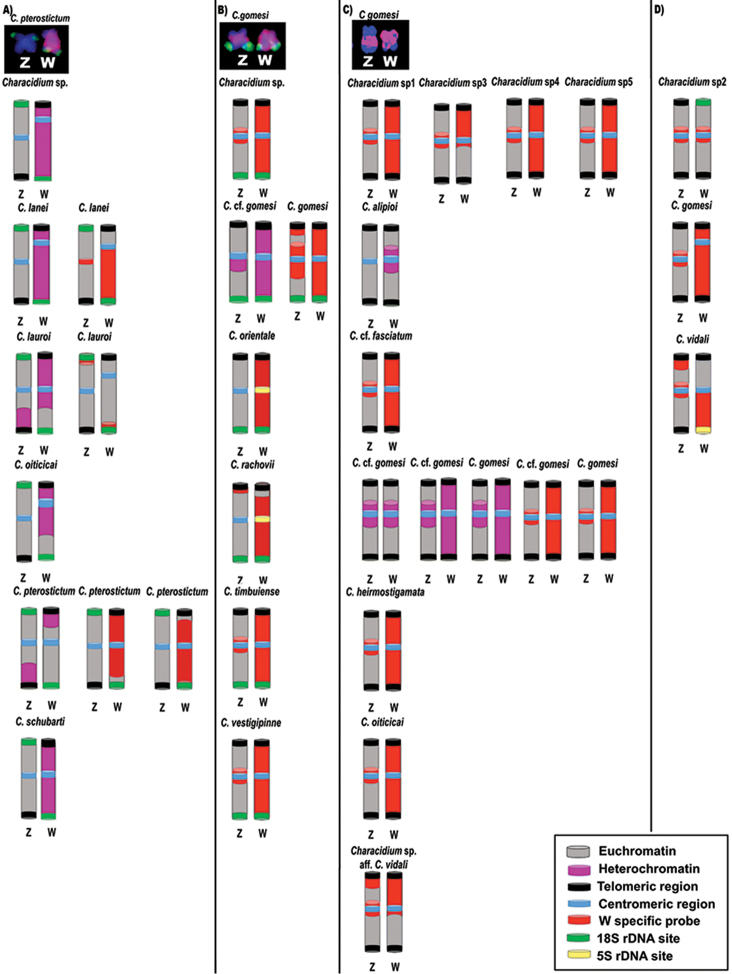
Idiograms showing main characteristics already identified for the ZZ/ZW sex chromosome system in *Characidium* species. It was highlighted the position of the centromere, distribution of euchromatin and heterochromatin, W-specific probes, and rDNA sites. The **a** column detaches the species carrying 18S rDNA sites on the short and long arms of the Z and W chromosomes, respectively; the **b** column highlights the species bearing 18S rDNA sites on the long arms of both Z and W chromosomes; the **c** column shows the species that do not present 18S rDNA sequences on either Z or W chromosomes; the **d** column presents the species bearing Z and W chromosomes with unusual characteristics, including morphology, 18S and 5S rDNA sites, and W-specific probe distribution.

Meiotic analyses revealed the bivalent pairing of the ZW chromosomes, as well as the bivalent plus one univalent formation in specimens carrying three B chromosomes (Serrano et al. 2016). Chromosome pairing does not always indicate complete homology between chromosomes (Ramsey and Schemske 2002). In fact, the Z and W sex chromosomes in *Characidium* species possesses differences in 45S rDNA chromosomal localization and in heterochromatin blocks extension (Fig. 3). Chromosomal localization differences of the repetitive sequences among *Characidium* species are also observed, such as in (TTA)_10_, (GAG)_10_, (CG)_15_ and (GATA)*_n_* sequences (Scacchetti et al. 2015b, Pucci et al. 2016). In *C.gomesi* it was shown that the short arm of the W chromosome keeps homology with the terminal region of the Z chromosome in relation to the (CG)_15_, (GATA)*_n_*, and (TAA)_10_ sequences (Pucci et al. 2016). (GATA)*_n_* and (TAA)_10_ homology is also present in the centromeric region of the *C.gomesi* (Pucci et al. 2016). These data help to explain ZW chromosome pairing and its bivalent formation in *Characidium* species.

The occurrence of a ZZ/ZW sex chromosome system is another karyotypic characteristic of *Characidium* genomes. It was first described by Maistro et al. (1998) in Characidiumcf.fasciatum Reinhardt, 1867 (Table 1), but it is also present in most *Characidium* species studied. The sex chromosomes in *Characidium* show a high degree of differentiation among species by chromosomal size, morphology, heterochromatin accumulation and presence or absence of rDNA sites (Maistro et al. 1998, 2004, Centofante et al. 2001, 2003, Vicari et al. 2008, Noleto et al. 2009, Pansonato-Alves et al. 2010, 2011b, 2014, Machado et al. 2011, Pazian et al. 2013, 2014, Pucci et al. 2014, 2016, Scacchetti et al. 2015a, 2015b, 2015c, Serrano et al. 2017), as exemplified in Fig. 3. Interestingly, the W chromosome can possess distinct cytotypes among *C.gomesi* populations, such as sm (Centofante et al. 2001, Pansonato-Alves et al. 2011b) or subtelocentric (Vicari et al. 2008, Pucci et al. 2014, 2016).

The majority of microsatellites sites were located in the terminal region of the Z chromosome and in the terminal/centromeric regions of W chromosome. The exception is (TTA)_10_, which was widely distributed throughout the whole W chromosome, and (GAG)_10_, which had a preferential accumulation in the W and B chromosomes of *C.alipioi* (Scacchetti et al. 2015b). (CG)_15_ and (GATA)*_n_* sequences were mainly found on the short arm of W chromosome in genomes of *C.zebra* and *C.gomesi*. It was suggested that these regions are enriched with sex-specific genes (Pucci et al. 2016), since the (GATA)*_n_* sequences are known as a motif for sex- and tissue-specific GATA-binding proteins. However, this pattern was not found in *Characidiumheirmostigmata* da Graça & Pavanelli, 2008 (Fig. 2).

18S rDNA sequences are also particular components of many *Characidium* sex chromosomes, occupying the short and the long arms of Z and W chromosomes, respectively, or the long arms of both sex chromosomes (Table 1, Fig. 3). These ribosomal sequences were likely associated with the origin of the protosex chromosome. It is likely that the NORs of the sm pair 23 (an ancestral pattern) were translocated to opposite arms of the second metacentric (m) pair (Machado et al. 2011, Pucci et al. 2014).

**Figure 4.**
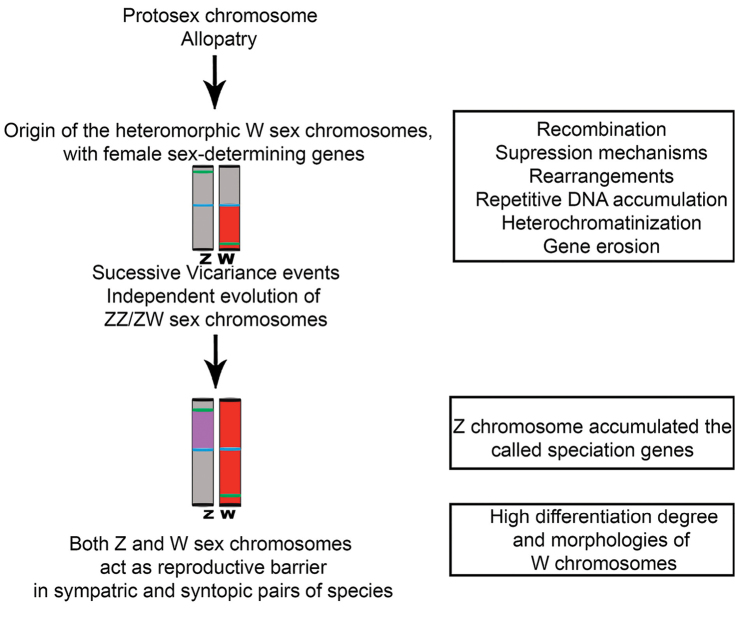
Schematic idiograms showing some steps proposed in the differentiation process of the ZZ/ZW sex pair. The origin of the ZZ/ZW sex pair from the protosex chromosome of the *Characidium* species. Centromeric region (blue); 18S rDNA site (green); W specific probe region (red); probable Z speciation genes region (purple).

Later differentiations in such protosex chromosomes were gradually acquired by isolated populations, leading to deletions and duplications in the rearranged regions due to meiotic pairing failures. Thus, recombination suppression mechanisms (rearrangements, heterochromatinization, repeated DNA accumulation and gene erosion) were naturally selected, giving rise to distinct heteromorphic W chromosomes (Machado et al. 2011, Pucci et al. 2014). Such modifications also promoted the accumulation of the so-called “speciation genes”, particularly in linked Z chromosome loci (Pucci et al. 2014). These genes established meiotic barriers and post-zygotic isolation mechanisms, along with the morphological variations of W chromosome (Fig. 4).

The current sympatric occurrence of some *Characidium* species does not display hybridization events among them. Sympatric and syntopic pairs of *Characidium* species, with the presence or absence of sex chromosomes, had already been described, namely *C.alipioi* and *Characidium* sp. cf. *C.lauroi* (Centofante et al. 2003), and C.cf.zebra and *C.gomesi* (da Silva and Maistro 2006). Thus, it is likely that NOR displacements throughout the genome was a key factor linked to W chromosome differentiation in Crenuchidae. Usually, when the W chromosome is partially heterochromatic, it is still a NOR bearing chromosome; but in totally heterochromatic chromosomes, NORs are found in different autosomes (Table 1, Fig. 3). Restriction-site associated DNA sequencing (RAD-seq) was applied to study the sex chromosomes of *C.gomesi* (Utsonomia et al. 2017). This application identifies 26 female-specific RAD loci, putatively located on the W chromosome, as well as 148 sex-associated SNPs showing significant differentiation. The use of W markers validated for *in situ* localization in other populations and species of the genus *Characidium* suggested a rapid turnover of W-specific repetitive elements (Utsonomia et al. 2017). This finding corroborates the inference that modifications on sex chromosomes also promote the accumulation of the “speciation genes”, leading to chromosomal speciation mechanisms in *Characidium*.

**Table 1.** Review of *Characidium* cytogenetic studies until 2018. The variation in the diploid number (2n) is due to the presence of B chromosomes. “Unknown” signifies that the data was not available in the original study. NOR: Nucleolar Organizer Region; M: Metacentric; SM: Submetacentric; ST: Subtelocentric; A: Acrocentric. * The chromosome pairs are not indicated in the original publication.

Species	Localization	2n	Sex chromosome system	Karyotype formula	rDNA 18S	rDNA 5S	References
*C.alipioi* Travassos, 1955	Ribeirão Grande Stream, SP, Brazil	50	ZZ/ZW	30M+20SM	Pair 16 (NOR)	Unknown	Centofante et al. (2003)
	Ribeirão Grande Stream, SP, Brazil	50–54	ZZ/ZW	32M+18SM	Pair 18	Pair 20	Serrano et al. (2017)
*C.fasciatum* Reinhardt, 1867	Rio São Francisco, MG, Brazil	50	ZZ/ZW	32M+18SM	Unknown	Unknown	Pazian et al. (2014)
C. cf. fasciatum	Rio das Velhas Stream, MG, Brazil	50	ZZ/ZW	Unknown	Unknown	Unknown	Pazian et al. (2013)
*C.gomesi* Travassos, 1956	Paiol Grande Stream, SP, Brazil	50	ZZ/ZW	♂ 32 M+18 SM	Pair 18	Unknown	Centofante et al. (2001)
				♀ 31 M+19SM			
*C.gomesi* (cited like C.cf.fasciatum)	Paranapanema, SP, Brazil	50–54	ZZ/ZW	32M+18SM	Three autossomic pairs*	Unknown	Maistro et al. (1998)
* C. gomesi *	Pardo River, SP, Brazil	50–54	ZZ/ZW	32M+18SM	Pair 17 and an additional chromosome (NOR)	Unknown	Maistro et al. (2004), Serrano et al. (2016)
	Machado River, MG, Brazil	50	Absent	32M+18SM	Pair 17 (NOR)	Unknown	da Silva and Maistro (2006)
C. cf. gomesi	Quebra Perna Stream, PR, Brazil	50	ZZ/ZW	♂ 32 M+18 SM	Pairs 4, 7 and 17	One autosomal pair*	Vicari et al. (2008), Pucci et al. (2014),.
				♀31M+18SM+1ST			
	Alambari Stream, SP, Brazil	50	ZZ/ZW	♂ 32 M+18 SM	ZW	Pairs 20 and 25	Machado et al. (2011)Pansonato-Alves et al. (2011b), Pazian et al. (2014)
				♀ 31 M+19SM			
	Novo River, SP, Brazil	50–54	ZZ/ZW	♂ 32 M+18 SM	Pair 18	Pair 25	Pansonato-Alves et al. (2011b, 2014)
				♀ 31 M+19SM			
* C. gomesi *	Verde River, PR, Brazil	50	ZZ/ZW	♂ 32 M+18 SM	Pairs 17, 22 and in one of the homologous of the pairs 1 and 20	Unknown	Machado et al. (2011)
				♀31+18SM+1ST			
C. cf. gomesi	Rio da Cachoeira Stream, GO, Brazil	50	ZZ/ZW	32M+18SM	Unknown	Unknown	Pazian et al. (2013, 2014)
	Magdalena Stream, SP, Brazil	50–52	ZZ/ZW	32M+18SM	Unknown	Unknown	Pazian et al. (2014)
* C. gomesi *	Grande River, SP, Brazil	50	ZZ/ZW	32M+18SM	Pair 17	Unknown	Machado et al. (2011)
	Minhoca Stream, MG, Brazil	50	ZZ/ZW	32M+18SM	Pair 17	Unknown	Machado et al. (2011)
	Tietê River, SP, Brazil	50	ZZ/ZW	32M+18SM	ZW	Unknown	Pansonato-Alves et al. (2014)
	São Domingos River, MG, Brazil	50	ZZ/ZW	32M+18SM	Pair 17	Unknown	Pansonato-Alves et al. (2014)
	Vermelho River, MT, Brazil	50	ZZ/ZW	32M+18SM	Pair 17	Unknown	Pansonato-Alves et al. (2014)
	São João River, PR, Brazil	50	ZZ/ZW	♂ 32 M+18 SM	Pairs 10 and 17	Unknown	Pucci et al. (2016)
				♀31M+18SM+1ST			
*C.heirmostigmata* da Graça & Pavanelli, 2008	Barra Grande River, PR, Brazil	50	ZZ/ZW	32M+18SM	Pair 4	Pair 19	Pucci et al. (2014)
*C.lagosantense* Travassos, 1947	Amendoim Stream, MG, Brazil	50	Absent	Unknown	Unknown	Unknown	Pazian et al. (2013)
C. cf. lagosantense	Infernao Lagoon, SP, Brazil	50	Unknown	32M+18SM	Unknown	Unknown	Miyazawa and Galetti (1994)
*C.lanei* Travassos, 1967	Barroca River, PR, Brazil	50	ZZ/ZW	32M+16SM+2A	ZW	One autosomal pair*	Noleto et al. (2009)
	Cari Stream, PR, Brazil	50	ZZ/ZW	32M+18SM	ZW (NOR)	One autosomal pair*	Pansonato-Alves et al. (2010), Scacchetti et al. (2015b, c),
*C.lauroi* Travassos, 1949	Grande River, SP, Brazil	50	ZZ/ZW	♂ 32 M+18 SM	ZW (NOR)	Unknown	Centofante et al. (2003)Pansonato-Alves et al. (2010), Machado et al. (2011)
				♀31M+18SM+1ST			
*C.oiticicai* Travassos, 1967	Pairaitinguinha River, SP, Brazil	50–53	ZZ/ZW	32M+18SM	ZW (NOR)	Unknown	Pansonato-Alves et al. (2010, 2014)
*C.orientale* Buckup & Reis, 1997	Chasqueiro Stream, RS, Brazil	50	ZZ/ZW	32M+18SM	ZW	Pairs 1, 3, 5, 6, 20 and W	Scacchetti et al. (2015a)
*C.pterostictum* Gomes, 1947	Betari River, SP, Brazil	50–53	ZZ/ZW	32M+16SM+2A	ZW	Unknown	Pansonato-Alves et al. (2010, 2014)
	Faú River, SP, Brazil	50	ZZ/ZW	32M+16SM+2A	ZW	Unknown	Pansonato-Alves et al. (2014)
	Cari River, PR, Brazil	50	ZZ/ZW	32M+16SM+2A	ZW	Unknown	Pansonato-Alves et al. (2014)
	Jacareí River, PR, Brazil	50	ZZ/ZW	32M+16SM+2A	ZW	Unknown	Pansonato-Alves et al. (2014)
	Itapocu River, SC, Brazil	50	ZZ/ZW	32M+16SM+2A	ZW	Unknown	Pansonato-Alves et al. (2014)
	Pairiquera-Açú River, SP, Brazil	50	ZZ/ZW	32M+16SM+2A	ZW	Pairs 9, 11 and 13	Pucci et al. (2014)
	Jacuí River, RS, Brazil	50	ZZ/ZW	32M+16SM+2A	ZW	Three autosomal pairs*	Scacchetti et al. (2015b)
	Itapeva Lagoon, RS, Brazil	50	ZZ/ZW	32M+16SM+2A	Unknown	Unknown	Scacchetti et al. (2015c)
	Carlos Botelho Ecological Station, SP, Brazil	50	Unknown	32M+16SM+2ST	Unknown	Unknown	Miyazawa and Galetti (1994)
*C.rachovii* Regan, 1913	Cabeças Stream, RS, Brazil	50	ZZ/ZW	32M+18SM	ZW	Pairs 1, 3 ,5, 17, 20 and W	Scacchetti et al. (2015a)
*C.schubarti* Travassos, 1955	Cinco Réis River, PR, Brazil	50	ZZ/ZW	32M+18SM	ZW (NOR)	Unknown	Pansonato-Alves et al. (2010), Scacchetti et al. (2015c)
*C.serrano* Buckup & Reis, 1997	Canoinha Stream, RJ, Brazil	50	ZZ/ZW	32M+16SM+2A	Unknown	Unknown	Scacchetti et al. (2015c)
*C.stigmosum* Melo & Buckup, 2002	Ave Maria River, GO, Brazil	50	Absent	32M+18SM	Pair 23	Pairs 1, 7 and 17	Scacchetti et al. (2015a)
*C.tenue* (Cope, 1894)	Chuí Stream, SC, Brazil	50	Absent	32M+18SM	Pair 23	Pairs 1 and 7	Scacchetti et al. (2015a)
*C.timbuiense* Travassos, 1946	Valsugana Velha Stream, ES, Brazil	50	ZZ/ZW	32M+16SM+2A	ZW	Three autosomal pairs*	Scacchetti et al. (2015b)
*C.vestigipinne* Buckup & Hahn, 2000	Caraguatá River, RS, Brazil	50	ZZ/ZW	32M+18SM	ZW	Pairs 1, 17 and 20	Scacchetti et al. (2015a)
*C.vidali* Travassos, 1967	Bananeiras Stream, RJ, Brazil	50	ZZ/ZW	32M+18SM	One autosomal pair*	W chromosome and in one autosomal pair*	Scacchetti et al. (2015b, c)
C. aff. vidali	Bananeiras Stream, RJ, Brazil	50–54	ZZ/ZW	32M+18SM	Pair 21	Pairs 5, 12 and 20	Scacchetti et al. (2015a)
*C.xavante* da Graça, Pavanelli & Buckup, 2008	Xingu River, MT, Brazil	50	Absent	32M+18SM	Pair 23	Pairs 1, 7 and 17	Scacchetti et al. (2015a)
*C.zebra* Eigenmann, 1909	Jatai Reservoir, SP, Brazil	50	Unknown	32M+18SM	Pair 25 (NOR), with 1 to 2 additional pairs	Unknown	Miyazawa and Galetti (1994)
C. cf. zebra	Passa Cinco River, SP, Brazil	50	Unknown	32M+18SM	Pair 23	Pair 17	Miyazawa and Galetti (1994)Machado et al. (2011), Pucci et al. (2014)
	Passa Cinco River, SP, Brazil	50–51	Unknown	Unknown	Unknown	Unknown	Venere et al. (1999)
	Piracicaba River, SP, Brazil	50	Unknown	32M+18SM	Pair 25 (NOR)	Unknown	Miyazawa and Galetti (1994)
	Ribeirão Claro Stream, SP, Brazil	50	Absent	Unknown	Unknown	Unknown	Pazian et al. (2013)
	Pairaitinga River, SP, Brazil	50	Absent	32M+18SM	Pair 23	Pairs 1, 6, and 17	Pansonato-Alves et al. (2010, 2011a), Scacchetti et al. (2015b, 2015c)
	Paiol Grande Stream, SP, Brazil	50	Absent	32M+18SM	Pair 23 (NOR)	Unknown	Centofante et al. (2001), Pucci et al. (2016)
	Machado River, MG, Brazil	50	Absent	32M+18SM	Pair 23 (NOR)	Unknown	da Silva and Maistro (2006)
	Alambari River, SP, Brazil	50	Absent	32M+18SM	Pair 23	Pair 17	Pansonato-Alves et al. (2011a)
	Novo River, SP, Brazil	50	Absent	32M+18SM	Pair 23	Pair 17	Pansonato-Alves et al. (2011a)
	Araquá River, SP, Brazil	50	Absent	32M+18SM	Pair 23	Pair 17	Pansonato-Alves et al. (2011a)
	Duas Antas Stream, MT, Brazil	50	Absent	32M+18SM	Pair 23	Pairs 1 and 17	Scacchetti et al. (2015a)
	Juba River, MT, Brazil	50	Absent	32M+18SM	Pair 23	Pairs 1, 6, 9, 17 and 18	Pansonato-Alves et al. (2011a)
C. aff. zebra	Corredeira Stream, SP, Brazil	50	Absent	32M+18SM	Pairs 4, 7 and 23	Pair 17	Pucci et al. (2014)
	Corredeira Stream, SP, Brazil	50	Absent	32M+18SM	Pairs 2, 4, 7, 20, 23 and 17	Pair 17	Pucci et al. (2014)
*Characidium* sp.	Preto River, SP, Brazil	50	ZZ/ZW	32M+18SM	ZW (NOR)	Unknown	Pansonato-Alves et al. (2010)
	Lagoon of the Corredeira Stream, SP, Brazil	50	ZZ/ZW	32M+16SM+2A	ZW	Pairs 3, 7, 8, 23 and 24	Pucci et al. (2014)
*Characidium* sp.2	Vermelho River, MT, Brazil	50	ZZ/ZW	32M+18SM	W and pair 7	Pair 17	Scacchetti et al. (2015a)
*Characidium* sp.	Formoso River, GO, Brazil	50	ZZ/ZW	32M+18SM	Unknown	Unknown	Pazian et al. (2013, 2014)
	Inferno Lagoon, SP, Brazil	50	Unknown	32M+18SM	Unknown	Unknown	Miyazawa and Galetti (1994)
*Characidium* sp.1	Russo River, MT, Brazil	50	ZZ/ZW	32M+18SM	Pair 7	Pair 17	Scacchetti et al. (2015a)
*Characidium* sp.3	Arinos River, MT, Brazil	50	ZZ/ZW	32M+18SM	Pair 1	Pair 1	Scacchetti et al. (2015a)
*Characidium* sp.4	Nanay River, Peru	50	ZZ/ZW	32M+18SM	Pair 7	Pair 18	Scacchetti et al. (2015a)
*Characidium* sp.5	Canoinha Stream, RS, Brazil	50	ZZ/ZW	32M+18SM	Pair 19	Pairs 1, 5 and 6	Scacchetti et al. (2015a)

## Perspectives on Characidium investigations

Fish cytogenetic and molecular studies have improved over the last few years, especially with regard to better identification of the karyotypic evolution and sex chromosome differentiation among different groups of fish, as well as genes or specific regions related to sex determination. W-specific repetitive probes were already constructed for *Characidium* using microdissection from female metaphase chromosomes and degenerate oligonucleotide-primed PCR (DOP-PCR) or whole genome amplification (WGA) protocols. These probes were later applied to chromosome painting in *Characidium* using a *C.gomesi* W-specific probe (Machado et al. 2011, Pazian et al. 2013, 2014, Pansonato-Alves et al. 2014, Pucci et al. 2014). This was followed by investigations of homologous regions between the sex pairs, B chromosomes and autosomes (Machado et al. 2011, Pazian et al. 2013, 2014, Pansonato-Alves et al. 2014, Pucci et al. 2014, 2016, Scacchetti et al. 2015a, 2015b, Serrano et al. 2016, 2017), and the cloning of a W-specific sequence that generated the CgW9 clone, which is similar to the zebrafish *Helitron* transposon (Pazian et al. 2014).

The ZZ/ZW sex chromosome system is well-known and described. The repeated DNA classes related to gene erosion and differentiation of W chromosome, as well as regions or genes implicated in sex determination and gonadal differentiation, have not yet been properly investigated in most species. It has been demonstrated that the repeated DNA sequences are closely related to the regulatory genes network, particularly TEs, in a process called molecular co-option or exaptation (Feschotte 2008). In this sense, future studies concerning the dynamics of mobile elements and molecular co-option in the regulatory system of *Characidium* will be relevant contributions to this research area. Sequencing and comparisons between male and female genomes of different *Characidium* species will contribute to highlighting the genic and/or repetitive sequences that are sex-restricted.

In other pathways, sequencing procedures of particular W fractions is needed for investigating specific genes related to sex determination and differentiation. Indeed, integrating cytogenetic, genomic, molecular, and bioinformatic tools will be essential for a better understanding of sex determination and differentiation processes in fishes, with applications in ecological and evolutionary studies.

## Conclusion

Chromosomal diversification in *Characidium* here revised show a diversified karyotype microstructure despite its conserved karyotypic macrostructure with prevalent 2n of 50 chromosomes arranged in 32 m + 18 sm. Differences in the number of rDNA sites, in heterochromatin blocks, in B chromosomes number and, in sex chromosomes sizes, as well as an interesting dynamic of repetitive DNAs on the chromosomes are observed among species, leading to chromosomal diversification and speciation. The data showed that different microsatellite expansions are involved in the sex chromosome differentiation in *Characidium*. In addition, the microsatellite (TTA)_10_ play an important role in gene degeneration and erosion on the W chromosome in some *Characidium* species. These data are important for the molecular characterization of the W and B chromosomes, to karyotype structures determination and comprehension of cryptic species. Future studies integrating cytogenetic, genomic and molecular data open perspectives to understand the sex determination, B chromosome composition and, “speciation genes” in *Characidium* genomes.

## References

[B1] Almeida-Toledo LF Foresti F Péquignot EV Daniel-Silva MFZ (2001) XX:XY sex chromosome system with X heterochromatinization: an early stage of sex chromosome differentiation in the Neotropic electric eel *Eigenmanniavirescens*. Cytogenetics and Cell Genetics 95: 73–78. 10.1159/000057020 11978973

[B2] Aranha JMR Gomes JHC Fogaça FNO (2000) Feeding of two sympatric species of *Characidium*, *C.lanei* and *C.pterostictum* (Characidiinae) in a coastal stream of Atlantic Forest (Southern Brazil). Brazilian Archives of Biology and Technology 43: 527–531. 10.1590/S1516-89132000000500013

[B3] Barbosa P Pucci MB Nogaroto V Almeida MC Artoni RF Vicari MR (2017) Karyotype analysis of three species of *Corydoras* (Siluriformes: Callichthyidae) from southern Brazil: rearranged karyotypes and cytotaxonomy. Neotropical Ichthyology 15: e160056. 10.1590/1982-0224-20160056

[B4] Bastos RF Miranda SF Garcia AM (2013) Dieta e estratégia alimentar de *Characidiumrachovii* (Characiformes, Crenuchidae) em riachos de planície costeira do sul do Brazil. Iheringia 103: 335–341. 10.1590/S0073-47212013000400001

[B5] Buckup PA (1993) Phylogenetic interraletionships and reductive evolution in neotropical characidiin fishes (Characiformes, Ostariophysi). Cladistics 9: 305–341. 10.1111/j.1096-0031.1993.tb00227.x 34929956

[B6] Buckup PA (1998) Relationships of the Characidiinae and phylogeny of characiform fishes (Teleostei: Ostariophysi). In: Malabarba LR, Reis RE, Vari RP, et al. (Eds) Phylogeny and classification of neotropical fishes. Edipucrs, Porto Alegre, 123–144.

[B7] Buckup PA (1999) Ecologia de peixe de riachos. Série Oecologia Brasiliensis, vol. VI. PPGE-UFRJ. In: Caramaschi EP Mazzoni R Peres Neto PR (Eds) Chapter 3, Sistemática e Biogeografia de Peixes de Riachos.Rio de Janeiro, 91–138.

[B8] Camacho JP Sharbel TF Beukeboom LW (2000) B-chromosome evolution. Philosophical Transactions of the Royal Society B: Biological Sciences 355: 163–178. 10.1098/rstb.2000.0556 PMC1692730 10724453

[B9] Carvalho PA Martins-Santos IC Dias AL (2008) B-chromosomes: an update about its occurrence in freshwaters Neotropical fishes (Teleostei). Journal of Fish Biology 72: 1907–1932. 10.1111/j.1095-8649.2008.01835.x

[B10] Centofante L Bertollo LAC Buckup PA Moreira-Filho O (2003) Chromosomal divergence and maintenance of sympatric *Characidium* fish species (Crenuchidae, Characidiinae). Hereditas 138: 213–218. 10.1034/j.1601-5223.2003.01714.x 14641486

[B11] Centofante L Bertollo LAC Moreira-Filho O (2001) Comparative cytogenetics among sympatric species of *Characidium* (Pisces, Characiformes). Diversity analysis with the description of a ZW sex chromosome system and natural triploidy. Caryologia 54: 253–260. 10.1080/00087114.2001.10589233

[B12] da Silva AR Maistro EL (2006) Cytogenetic divergence between two sympatric species of *Characidium* (Teleostei, Characiformes, Crenuchidae) from the Machado River, Minas Gerais, Brazil. Genetics and Molecular Biology 29: 459–463. 10.1590/S1415-47572006000300010

[B13] do Nascimento VD Coelho KA Nogaroto V Almeida RB Ziemniczak K Centofante L Pavanelli CS Torres RA Moreira-Filho O Vicari MR (2018) Do multiple karyomorphs and population genetics of freshwater darter characines (*Apareiodonaffinis*) indicate chromosomal speciation? Zoologischer Anzeiger 272: 93–103. 10.1016/j.jcz.2017.12.006

[B14] Eschmeyer WN Fricke R van der Laan R (Eds) (2018) Catalog of fishes: genera, species, references [Internet]. http://researcharchive.calacademy.org/research/ichthyology/catalog/fishcatmain.asp [updated 2018 Apr 30, cited 2018 May 21]

[B15] Fernandes S Leitão RF Dary EP Guerreiro AIC Zuanon J Bührnheim CM (2017) Diet of two syntopic species of Crenuchidae (Ostariophysi: Characiformes) in an Amazonian rocky stream. Biota Neotropica 17(1): e20160281. 10.1590/1676-0611-bn-2016-0281

[B16] Feschotte C (2008) Transposable elements and the evolution of regulatory networks. Nature Reviews Genetics 9: 397–405. 10.1038/nrg2337 PMC2596197 18368054

[B17] Glugoski L Giuliano-Caetano L Moreira-Filho O Vicari MR Nogaroto V (2018) Co-located hAT transposable element and 5S rDNA in an interstitial telomeric sequence suggest the formation of Robertsonian fusion in armored catfish. Gene 650: 49–50. 10.1016/j.gene.2018.01.099 29408629

[B18] Machado TC Pansonato-Alves JC Pucci MB Nogaroto V Almeida MC Oliveira C Foresti F Bertollo LAC Moreira-Filho O Vicari MR (2011) Chromosomal painting and ZW sex chromosomes differentiation in *Characidium* (Characiformes, Crenuchidae). BMC Genetics 12: 65. 10.1186/1471-2156-12-65 PMC3160954 21787398

[B19] Maistro EL de Jesus CM Oliveira C Moreira-Filho O Foresti F (2004) Cytogenetic analysis of A-, B-chromosomes and ZZ/ZW sex chromosomes of *Characidiumgomesi* (Teleostei, Characiformes, Crenuchidae). Cytologia 69: 181–186. 10.1508/cytologia.69.181

[B20] Maistro EL Mata EP Oliveira C Foresti F (1998) Unusual occurrence of a ZZ/ZW sex-chromosome system and supernumerary chromosomes in Characidiumcf.fasciatum (Pisces, Characiformes, Characidiinae). Genetica 104: 1–7. 10.1023/A:1003242020259 16220371

[B21] Matsunaga S (2009) Junk DNA promotes sex chromosome evolution. Heredity 102: 525–526. 10.1038/hdy.2009.36 19337304

[B22] Melo MRS Oyakawa OT (2015) A new species of *Characidium* Reinhardt (Characiformes, Crenuchidae) with a distinctively dimorphic male. Copeia 103: 281–289. 10.1643/CI-14-073

[B23] Miyazawa CS Galetti Jr PM (1994) First cytogenetical studies in *Characidium* species (Pisces: Characiformes, Characidiinae). Cytologia 59: 73–79. 10.1508/cytologia.59.73

[B24] Moreira-Filho O Bertollo LAC Galetti Jr PM (1993) Distribution of sex chromosome mechanisms in Neotropical fish and description of a ZZ/ZW system in *Parodonhilarii* (Parodontidae). Caryologia 46: 115–125. 10.1080/00087114.1993.10797253

[B25] Noleto RB Amorim AP Vicari MR Artoni RF Cestari MM (2009) An unusual ZZ/ZW sex chromosome system in *Characidium* fishes (Crenuchidae, Characiformes) with the presence of rDNA sites. Journal of Fish Biology 75: 448–453. 10.1111/j.1095-8649.2009.02342.x 20738550

[B26] Pansonato-Alves JC Oliveira C Foresti F (2011a) Karyotypic conservatism in samples of Characidiumcf.zebra (Teleostei, Characiformes, Crenuchidae): Physical mapping of ribosomal genes and natural triploidy. Genetics and Molecular Biology 34: 208–213. 10.1590/S1415-47572011005000005 21734818 PMC3115311

[B27] Pansonato-Alves JC Paiva LRS Oliveira C Foresti F (2010) Interespecific chromosomal divergences in the genus *Characidium* (Teleostei: Characiformes: Crenuchidae). Neotropical Ichythyology 8: 77–86. 10.1590/S1679-62252010000100010

[B28] Pansonato-Alves JC Serrano EA Utsunomia R Camacho JPM Silva GJC Vicari MR Artoni RF Oliveira C Foresti F (2014) Single origin of sex chromosomes and multiple origins of B chromosomes in fish genus *Characidium* Plos One 9: e107169. 10.1371/journal.pone.0107169 PMC4165761 25226580

[B29] Pansonato-Alves JC Vicari MR Oliveira C Foresti F (2011b) Chromosomal diversification in populations of Characidiumcf.gomesi (Teleostei, Crenuchidae). Journal of Fish Biology 78: 183–194. 10.1111/j.1095-8649.2010.02847.x 21235554

[B30] Pazian MF Oliveira C Foresti F (2014) Sex chromosome composition revealed in *Characidium* fishes (Characiformes: Crenuchidae) by molecular cytogenetic methods. Biologia 69: 1410–1416. 10.2478/s11756-014-0434-0

[B31] Pazian MF Shimabukuro-Dias CK Pansonato-Alves JC Oliveira C Foresti F (2013) Chromosome painting of Z and W sex chromosomes in *Characidium* (Characiformes, Crenuchidae). Genetica 141: 1–9. 10.1007/s10709-013-9701-1 23344657

[B32] Poveda-Martínez D Sosa CC Chacón-Vargas K García-Merchán VH (2016) Historical biogeography of five *Characidium* fish species: Dispersal from the amazon paleobasin to southeastern South America. PLoS One 11: e0164902. 10.1371/journal.pone.0164902 PMC5065214 27741308

[B33] Pucci MB Barbosa P Nogaroto V Almeida MC Artoni RF Pansonato-Alves JC Foresti F Moreira-Filho O Vicari MR (2014) Population differentiation and speciation in the genus *Characidium* (Characiformes: Crenuchidae): effects of reproductive and chromosomal barriers. Biological Journal of Linnean Society 111: 541–553. 10.1111/bij.12218

[B34] Pucci MB Barbosa P Nogaroto V Almeida MC Artoni RF Pansonato-Alves JC Scacchetti PC Foresti F Moreira-Filho O Vicari MR (2016) Chromosomal spreading of microsatellites and (TTAGGG)*_n_* sequences in the *Characidiumzebra* and *C.gomesi* genomes (Characiformes: Crenuchidae). Cytogenetic and Genome Research 149: 182–190. 10.1159/000447959 27504623

[B35] Pucci MB Nogaroto V Moreira-Filho O Vicari MR (2018) Dispersion of transposable elements and multigene families: Microstructural variation in *Characidium* (Characiformes: Crenuchidae) genomes. Genetics and Molecular Biology 41: 585–592. 10.1590/1678-4685-GMB-2017-0121 30043833 PMC6136364

[B36] Ramsey J Schemske DW (2002) Neopolyploidy in flowering plants. Review of Ecology and Systematics 33(1): 589–639. 10.1146/annurev.ecolsys.33.010802.150437

[B37] Scacchetti PC Utsunomia R Pansonato-Alves JC Vicari MR Artoni RF Oliveira C Foresti F (2015b) Chromosomal mapping of repetitive DNAs in *Characidium* (Teleostei, Characiformes): Genomic organization and diversification of ZW sex chromosomes. Cytogenetic and Genome Research 146: 136–143. 10.1159/000437165 26277929

[B38] Scacchetti PC Utsunomia R Pansonato-Alves JC Silva GJC Vicari MR Artoni RF Oliveira C Foresti F (2015a) Repetitive DNA sequences and evolution of ZZ/ZW sex chromosomes in *Characidium* (Teleostei: Characiformes). PLoS One 10: e0137231. 10.1371/journal.pone.0137231 PMC4570811 26372604

[B39] Scacchetti PC Utsunomia R Pansonato-Alves JC da Costa-Silva GJ Oliveira C Foresti F (2015c) Extensive spreading of interstitial telomeric sites in the chromosomes of *Characidium* (Teleostei, Characiformes). Genetica 143: 263–270. 10.1007/s10709-014-9812-3 25547849

[B40] Schemberger MO Bellafronte E Nogaroto V Almeida MC Schühli GS Artoni RF Moreira-Filho O Vicari MR (2011) Differentiation of repetitive DNA sites and sex chromosome systems reveal closely related group in Parodontidae (Actinopterygii: Characiformes). Genetica 139: 1499–1508. 10.1007/s10709-012-9649-6 22527690

[B41] Schemberger MO Oliveira JIN Nogaroto V Almeida MC Artoni RF Cestari MM Moreira-Filho O Vicari MR (2014) Construction and characterization of a repetitive DNA library in Parodontidae (Actinopterygii: Characiformes): A genomic and evolutionary approach to the degeneration of the W sex chromosome. Zebrafish 11: 518–527. 10.1089/zeb.2014.1013 25122415 PMC4248244

[B42] Serrano EA Araya-Jaime C Suárez-Villota EY Oliveira C Foresti F (2016) Meiotic behavior and H3K4m distribution in B chromosomes of *Characidiumgomesi* (Characiformes, Crenuchidae). Comparative Cytogenetics 10: 255–268. 10.3897/CompCytogen.v11i1.10886 27551347 PMC4977801

[B43] Serrano EA Utsunomia R Scudeller OS Oliveira C Foresti F (2017) Origin of B chromosomes in *Characidiumalipioi* (Characiformes, Crenuchidae) and its relationship with supernumerary chromosomes in other *Characidium* species. Comparative Cytogenetics 11: 81–95. 10.3897/CompCytogen.v11i1.10886 28919951 PMC5599694

[B44] Utsunomia R Scacchetti PC Hermida M Fernández-Cebrián R Taboada X Fernández C Bekaert M Mendes NJ Robledo D Mank JE Taggart JB Oliveira C Foresti F Martínez P (2017) Evolution and conservation of *Characidium* sex chromosomes. Heredity 119: 237–244. 10.1038/hdy.2017.43 28745717 PMC5597785

[B45] Vicari MR Artoni RF Moreira-Filho O Moreira-Filho O (2008) Diversification of a ZZ/ZW sex chromosome system in *Characidium* fish (Crenuchidae, Characiformes). Genetica 134: 311–317. 10.1007/s10709-007-9238-2 18175199

[B46] Vicari MR Pistune HFM Castro JP Almeida MC Bertollo LAC Moreira-Filho O Camacho JPM Artoni RF (2011) New insights on the origin of B chromosomes in *Astyanaxscabripinnis* obtained by chromosome painting and FISH. Genetica 139: 1073–1081. 10.1007/s10709-011-9611-z 21948070

[B47] Zanata AM Camelier P (2015) Two new species of *Characidium* Reinhardt (Characiformes: Crenuchidae) from northeastern Brazilian coastal drainages. Neotropical Ichthyology 13: 487–498. 10.1590/1982-0224-20140106

[B48] Zanata AM Ohara WM (2015) A new species of *Characidium* Reinhardt (Ostariophysi: Characiformes: Crenuchidae) from headwaters of rio Pacaás Novos, rio Madeira basin, Rondônia, Brazil. Zootaxa 4021: 368–376. 10.11646/zootaxa.4021.2.7 26624134

[B49] Ziemniczak K Traldi JB Nogaroto V Almeida MC Artoni RF Moreira-Filho O Vicari MR (2014) In situ localization of (GATA)*_n_* and (TTAGGG)*_n_* repeat DNAs and W sex chromosome differentiation in Parodontidae (Actinopterygii: Characiformes). Cytogenetic and Genome Research 144: 325–332. 10.1159/000370297 25662193

